# Computational Comparative Genomics of Eu- and Prokaryotes

**DOI:** 10.1002/cfg.163

**Published:** 2002-04

**Authors:** Clemens Suter-Crazzolara, Günther Kurapkat

**Affiliations:** Lion bioscience Waldhoferstr. 98 Heidelberg 69123 Germany


**Comparative and Functional Genomics**
*Comp Funct Genom* 2002; **3**: 167–168.
Published online 12 March 2002 in Wiley InterScience (www.interscience.wiley.com). **DOI:** 10.1002/cfg.163

**Conference Review**

### Computational comparative genomics of eu- and prokaryotes

Clemens Suter-Crazzolara* and Günther Kurapkat
*Lion bioscience, Waldhoferstr. 98, 69123 Heidelberg, Germany*

**Correspondence to:*
*Lion bioscience, Waldhoferstr. 98, 69123 Heidelberg, Germany.*
*E-mail: suter@lionbioscience.com*

Received: 8 January 2002
Accepted: 14 February 2002

With the rapid advent of worldwide genome projects, scientists are confronted with a tremendous increase in the amount of novel information. However, the gap between data collection and interpretation is also growing. For this reason, genome databases contain a wealth of information which is accessible, but which may remain obscured.

Much can be learned from comparing different genomes, as genomes of distant organisms may still encode proteins with high sequence similarity. The order of genes (colinearity) in genomes may also be conserved to some extent. We have employed both these observations to create a multi-functional, computational analysis system (genomeSCOUT™), which allows for rapid identification and functional characterization of genes and proteins through genome comparison. Using a number of independent methods, information about different levels of protein homology (concerning e.g. orthologs and clusters of orthologous groups, COGs), as well as protein families and gene order, is collected and stored in several databases. These databases are then used for interactive comparison of genomes and subsequent analysis. genomeSCOUT™ was extensively used for the analysis of two *Listeria* genomes (Figure 1; [1]). A free demo version of the genomeSCOUT / *Listeria* server is accessible at: **http://GS-Listeria.lionbioscience.com/**.

genomeSCOUT™ can easily be extended to allow the analysis of any number of single or multiple,

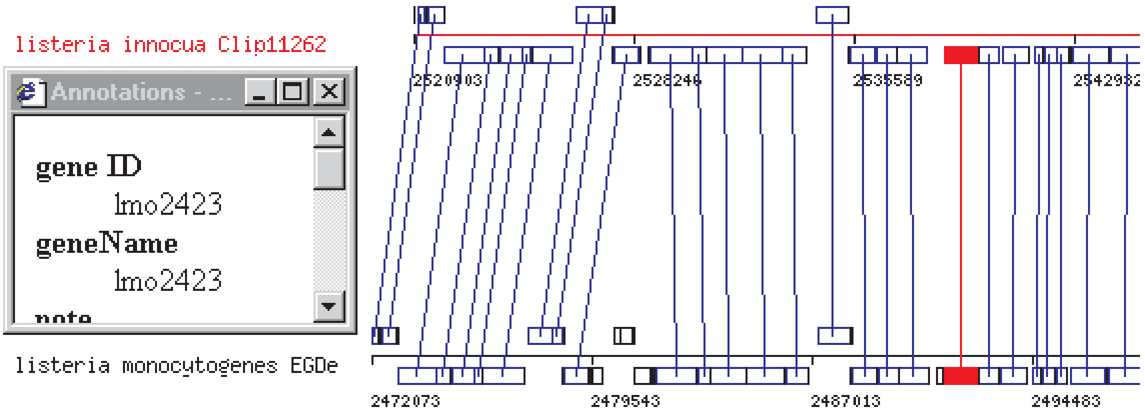

**Figure 1.** Rapid identification of unique and syntenic regions within two *Listeria* genomes, with genomeSCOUT™

Copyright © 2002 John Wiley & Sons, Ltd.

complete or partial, pro- or eukaryotic genomes. It is based on the well-established data integration system SRS. Its characteristics are, in addition to fast handling of large genomic data sets and straightforward access to a multitude of biological databases, the unique linking between these databases (**http://www.lionbioscience.com/**).

### References

1. Glaser P, Frangeul L, Buchrieser C, *et al.* 2001. *Comparative genomics of* Listeria *species. Science* **294**: 849–852.

Copyright © 2002 John Wiley & Sons, Ltd. 
